# Validity and Reliability of the Persian Version of COVID-19 Anxiety Syndrome Scale Among the Iranian General Population

**DOI:** 10.3389/fpubh.2022.845015

**Published:** 2022-06-15

**Authors:** Esmaeil Hoseinzadeh, Abbas Ebadi, Hamid Sharif Nia, Erika Sivarajan Froelicher, Pardis Rahmatpour

**Affiliations:** ^1^Department of Nursing, School of Nursing and Midwifery, Tehran Islamic Azad University of Medical Sciences, Tehran, Iran; ^2^Behavioral Sciences Research Center, Life Style Institute, Baqiyatallah University of Medical Sciences, Tehran, Iran; ^3^Nursing Faculty, Baqiyatallah University of Medical Sciences, Tehran, Iran; ^4^Department of Nursing, School of Nursing and Midwifery, Mazandaran University of Medical Sciences, Sari, Iran; ^5^Department of Nursing, School of Nursing and Midwifery Amol, Mazandaran University of Medical Sciences, Sari, Iran; ^6^Department of Physiological Nursing, School of Nursing, University of California, San Francisco, San Francisco, CA, United States; ^7^Department of Epidemiology & Biostatistics, School of Medicine, University of California, San Francisco, San Francisco, CA, United States; ^8^Department of Nursing, Alborz University of Medical Sciences, Karaj, Iran

**Keywords:** COVID-19, anxiety syndrome, Iran, validity, reliability

## Abstract

The crisis of the COVID-19 prevalence in Iran, as well as the world, caused mental disorders and anxiety syndrome. The COVID-19 anxiety syndrome scale (C-19ASS) assesses conceptually and psychometrically the nature of the COVID-19 threat experience instead of a response to the threat, fear, and COVID-19 anxiety. Therefore, the aim of this study is to evaluate the psychometric properties of the Persian version of the anxiety syndrome scale of COVID-19 in the population of Iran. The Persian version of C-19ASS was sent to Iranian adults *via* online social networking applications and finally, 932 adults responded to the questionnaire. The results of exploratory factor analysis revealed two-factor structures for C-19ASS, which explained 48.70% of the total variance. Given the confirmatory factor analysis findings, all goodness of fit indices confirmed the model fit. All coefficients of internal consistency were estimated as acceptable reliability. The results showed that the C-19ASS has good psychometric properties, and can be used by researchers, psychologists, and healthcare providers to assess the anxiety syndrome of the Iranian population during the COVID-19 pandemic.

## Introduction

In Iran, from 3 January 2020 to 15 March 2022, there have been 7,126,906 confirmed cases of COVID-19 with 139,063 deaths, according to WHO (1). It is well known that stressful life events can cause psychological symptoms (2). The public physical health, anxieties, and human safety are affected by this infectious disease that has caused numerous psychological health problems and psychological symptoms (3, 4). The COVID-19 outbreak is exacerbating the anxiety that many people feel. Anxiety is defined as distress or fear caused by the prediction of an event or a real or understandable threatening situation (3, 5). Anxiety syndrome may appear in a combination of avoidance, worrying, and monitoring of threat, such a set of incompatible contrasting forms may have an essential role in the stability of psychological depression (6).

The COVID-19 pandemic has exerted high pressures on health systems of countries where the virus is most prevalent (7). It has destructive and variable effects on all aspects of human life (8). It led to lockdowns that lasted over multiple months affecting educational and non-essential business activities in many countries. These lockdowns were implemented for the rapid reduction of COVID-19 transmission (9). All affected countries have a complete or partial cessation of social activities and a wide range of interventions. Examples include social isolation, individual isolation, social distancing, and quarantine to prevent the gathering of large numbers of people, beyond the immediate members of the household.

Many people who have been quarantined may feel lonely, bored, inactive, and insecure about food and economic issues as well as feel fear and anxiety about the infection caused by the disease (10). National polls show a severe increase of fear and anxiety about the virus (11). For example, a study of 44,000 participants in Belgium, conducted at the beginning of April 2020, reported that 20% of people had anxiety and 16% of them had a depressive disorder (11). Based on the model provided in Iranian study anxiety syndrome and fear of COVID-19 with mediation effects of perceived stress explained 70% of the total variance of psychological behavioral responses (4). According to the clinical manifestations of this disease such as respiratory failure, sepsis, shock, and various organ failures, it is understandable that medical professionals and public health specialists focus on caring for the sick people. While it is recognized that corona virus spreads to the general population, there is less attention given to the mental health consequences of the COVID-19 crisis (12). About 54%of the Chinese general population (*n* = 1,210) reported moderate or severe psychological effects of the disease outbreak, 16.5% reported moderate or severe depressive symptoms, and 28.8% reported moderate or severe anxiety symptoms (13). In the systematic review study of Salari et al. (14) the prevalence of stress, anxiety, and depression among the general population during the COVID-19 pandemic were 29.6% (sample size of 9,074), 31.9% (sample size of 63,439), and 33.7% (sample size of 44,531), respectively. Previous research has shown that people who suffer from pandemic anxiety tend to also show an increase in stress, anxiety, and suicide (15).

There are different scales such as the scale of the fear of COVID-19 (16), Coronavirus anxiety scale (17), scale of threat of Coronavirus (18), and COVID stress scale (19). These scales are intended to identify people who have been affected by anxiety, fear, and uncertainty over this growing epidemic crisis. However, the COVID-19 anxiety syndrome scale (C-19ASS) assesses conceptually and psychometrically the nature of the COVID-19 threat experience instead of a response to threat, fear, and COVID-19 anxiety. This scale has been developed and psychometrically tested by Nikcevic and Spada in the U.K. This scale identifies features of anxiety syndrome related to COVID-19 and assessed its validity and reliability (6).

Considering the prevalence of COVID-19 anxiety syndrome in Iran, and the lack of an accurate scale to measure it among the Iranian population, the aim of this study is to evaluate the psychometric properties of the Persian version of the anxiety syndrome scale of COVID-19 in the population of Iran.

## Methods

This methodological study design was used to achieve the research objective. Data collection took place between October and November 2021.

### Measurement

The measurements consist of two parts: demographic information, and the Persian version of C-19ASS. The original version of C-19ASS consists of nine items and two factors including perseveration (six items) and avoidance (three items). The C-19ASS is a short easily administered scale that can be used with both healthy and frail individuals exposed to any specific traumatic event. The response options for the C-19ASS are scored on a 5-point Likert-type scale from 0 (not at all) to 4 (Nearly every day) (6).

### Translation

At first, the written permission was obtained from the authors of the scale, Professor Marcantonio M. Spada *via* email. Then, two English-Persian translators translated the C-19ASS independently. The research team, as well as two professional translators, evaluated the two translations and created a Persian translation of C-19ASS. In the next step, two Persian to English translators who had no knowledge of the English version of the C-19ASS were asked to back-translate the Persian version of the C-19ASS scale into English. Then the panel of experts compiled and compared the results of the back-translation with the original instrument to detect any differences and similarities between the original instrument and the back-translated version.

All items translate into Persian and back-translated into English without any required modifications and Dr. Marcantonio M. Spada (The main developer scale) confirmed The Backward Scale. It is noteworthy that all the steps of this process were performed based on the World Health Organization protocol of forward-backward translation technique (20).

### Participants

The Persian version of C-19ASS was sent to Iranian adults *via* an online data gathering and 932 adults responded to the questionnaire. The online scale was created *via* Google Forms and its URL link was sent by email or social networking applications such as a Telegram channel or WhatsApp to a group of adults. To prevent duplicate data, the Google Form was restricted to get the data from each individual once. The inclusion criteria for participants were adults (age > 18) who were willing to participate in this study. The sample size should be at least 200 cases for factor analysis (21). Of these, 466 subjects were used for the exploratory factor analysis (EFA) and a second group with 466 subjects serves as the confirmatory factor analysis (CFA).

### Data Analysis

The construct validity of the Persian version of C-19ASS was evaluated by exploratory and confirmatory factor analysis. Maximum likelihood EFA with Promax rotation was conducted. The Kaiser–Meyer–Olkin test (KMO > 0.7: acceptable) and Bartlett's test of sphericity were calculated. The number of factors was determined based on parallel analysis, scree plot. Items with absolute loading values of 0.3 or greater and communalities more than 0.2 were considered appropriate (21). For assessment of the extracted factors, CFA was conducted using the maximum-likelihood method and the most common goodness of fit indices.

According to Fornell and Larcker's criteria (22), the Average Variance Extracted (AVE), Maximum Shared Squared Variance (MSV), and Composite Reliability (CR) were estimated to assess the convergent and discriminant validity. In addition, discriminant validity was evaluated by heterotrait-monotrait ratio of correlations (HTMT) approach. All values in the HTMT matrix table should be <0.85 (23). The reliability of the scale was evaluated using internal consistency and construct reliability (CR). The average inter-item correlation (AIC) was in the range of 0.2 to 0.4, Cronbach's alpha and McDonald's omega was >0.7 and are considered acceptable internal consistency (24). The CR was calculated using the structural equation model analysis as an alternative to Cronbach's alpha coefficient – it was acceptable if it was >0.7 (25). The relationship between demographic information and level of C-19ASS were evaluated by independent *t*-test, one-way ANOVA, and Pearson correlation coefficient.

#### Multivariate Normality and Outliers

Both univariate and multivariate normality of the data was evaluated in this study. The univariate distributions were tested for outliers, skewness, and kurtosis. The normality of the multivariate distribution was assessed using Mardia's coefficient of multivariate kurtosis, and the Mardia's coefficient. Mardia's coefficient > 7.98 can be considered as indicative of departure from multivariate normality. Moreover, the outliers of the multivariate distribution were detected using Mahalanobis distance (*P* < 0.001) (21).

The SPSS_26_, SPSS-R menu_2_, AMOS_26_, and JASP_0.15.0.0_ software were used to perform all of the statistical calculations.

### Ethical Consideration

The Tehran Islamic Azad University of Medical Sciences Research Ethics Committee approved the protocol of this study (IR.IAU.TMU.REC.1400.315). While sending the online scale through social networking programs, the objectives of the study were fully explained to the participants. Subjects were informed that participation was voluntary and that their decision would not affect their care. Participants were reassured about the confidentiality of the data.

## Results

The mean and standard deviation for the age of 932 adults was 31.14 (SD = 7.81) years. Other demographic characteristics of participants are shown in Table 1. Also, the level of C-19ASS was estimated 31.68 (SD = 8.23, CI 95%: 31.14–32.21).

**Table 1:** Demographic characteristics of participants (*n* = 932).

**Variables**	**EFA (466): *n* (%)**	**CFA (466): *n* (%)**
**Gender**	388 (83.26)	404(87.12)
Female	380 (81.54)	414 (88.84)
Male	78 (16.73)	60 (12.87)
**Marital status**		
Single	164 (35.19)	134 (28.75)
Married	302 (64.80)	332 (71.24)
**Education level**		
Under diploma	3 (0.64)	10 (2.14)
Diploma	19 (4.07)	34 (7.29)
Upper Diploma	26 (5.57)	59 (12.66)
Bachelor	233 (50)	207 (44.42)
Master	155 (33.26)	125 (26.82)
PhD	30 (6.43)	31 (6.65)

**Figure 1:**
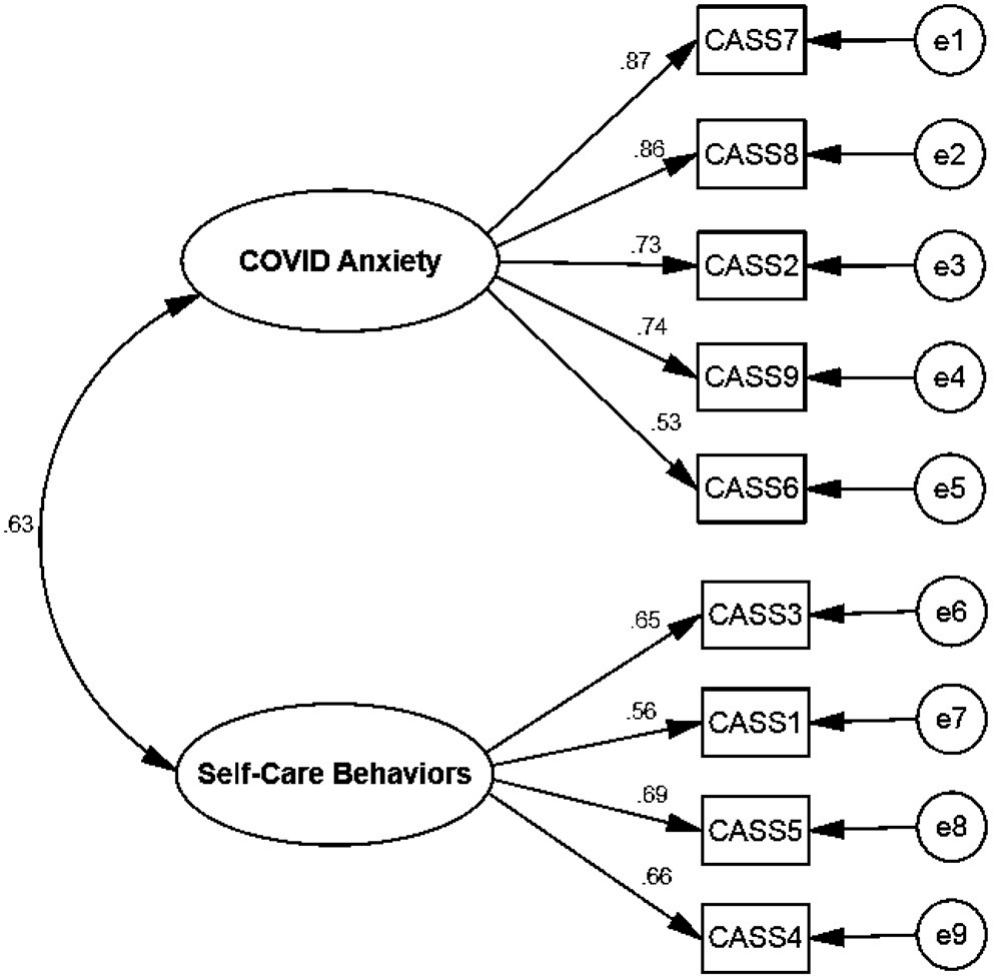
The CFA model of the C-19ASS.

In maximum likelihood EFA, the KMO test value was 0.852, and Bartlett's test value was 30,036.137 (*P* < 0.001). The EFA results revealed two factors with 48.70% explained variance for the C-19ASS (see Table 2, Figure 1).

**Table 2:** Exploratory factors extracted of COVID-19 anxiety syndrome scale (C-19ASS; *n* = 466).

**Factors**	**Q_**n**_. Item**	**Factor loading**	***h* ^2^**	**λ**	**%Variance**
**COVID-19 Anxiety**	**7**. I have checked my family members and loved one for the signs of coronavirus (COVID-19).	0.932	0.772	2.748	30.535
	**8**. I have been paying close attention to others displaying possible symptoms of coronavirus (COVID-19).	0.817	0.640		
	**2**. I have checked myself for symptoms of coronavirus (COVID-19).	0.706	0.519		
	**9**. I have imagined what could happen to my family members if they contracted coronavirus (COVID-19).	0.650	0.461		
	**6**. I have read about news relating to coronavirus (COVID-19) at the cost of engaging in work (such as writing emails, working on word documents or spreadsheets).	0.494	0.320		
**Self-care behaviors**	**3**. I have avoided going out to public places (shops, parks) because of the fear of contracting coronavirus (COVID-19).	0.707	0.498	1.636	18.173
	**1**. I have avoided using public transport because of the fear of contracting coronavirus (COVID-19).	0.641	0.341		
	**5**. I have avoided touching things in public spaces because of the fear of contracting coronavirus (COVID-19).	0.571	0.389		
	**4**. I have been concerned about not having adhered strictly to social distancing guidelines for coronavirus (COVID-19).	0.530	0.444		

*λ, Eigenvalue; h^2^, Communalities*.

**Figure 2:**
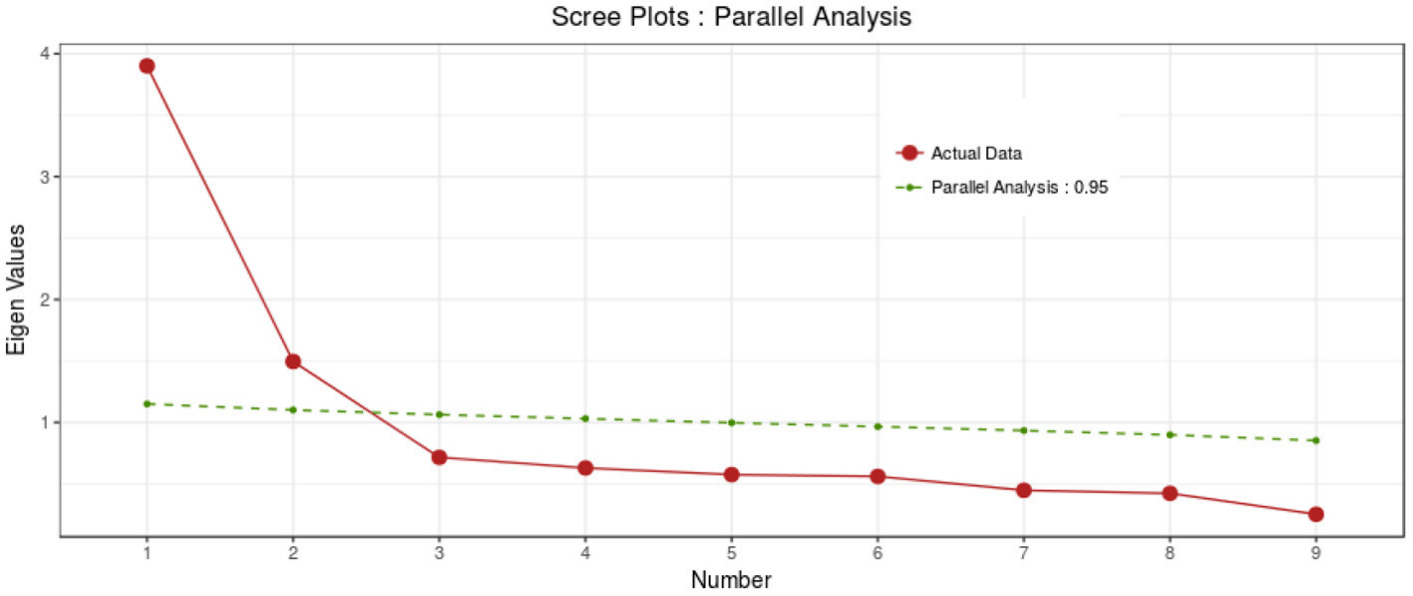
The scree plot of the C-19ASS.

The acceptable fit indices showed that the model was confirmed (see Table 3 and Figure 2). The convergent and discriminant validity for both factors were acceptable for the current study. The value in the HTMT matrix was <0.66, indicating discriminant validity was established in this study. All coefficients of internal consistency were estimated as acceptable reliability (see Table 4). There was no significant relationship between demographic variables and C-19ASS.

**Table 3:** Fit indices of the first order confirmatory factor analysis of the C-19ASS (*n* = 466).

**CFA index**	**CFI**	**IFI**	**PCFI**	**PNFI**	**RMSEA**	**CMIN/DF**	***P*-Value**	**df**	**χ^2^**
	0.957	0.957	0.691	0.672	0.033	2.107	<0.001	26	54.798

*DF, Degree of freedom; PCFI, Parsimonious Comparative Fit Index; PNFI, Parsimonious Normed Fit Index; CMIN/DF, Minimum Discrepancy Function divided by Degrees of Freedom; RMSEA, Root Mean Square Error of Approximation; TLI, Tuker-Lewis Index; and CFI, Comparative Fit Index, IFI, Incremental Fit Index, Fitness indexes, PNFI, PCFI (>0.5); TLI, IFI, CFI (>0.9), RMSEA (<0.08), CMIN/DF (<3 good, <5 acceptable)*.

**Table 4:** The indices of the convergent, discriminant validity, and internal consistency C-19ASS for the CFA (*n* = 466).

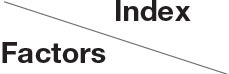	**CR**	**AVE**	**MSV**	**MaxR (*H*)**	**Alpha (CI95%)**	**Omega**	**AIC**
COVID-19 anxiety	0.867	0.573	0.399	0.897	0.844(0.828 to 0.859)	0.849	0.521
Self-care behaviors	0.738	0.417	0.399	0.743	0.721(0.691to 0.749)	0.726	0.396

## Discussion

The results of this study demonstrated that the Persian version of C-19ASS has nine items in two subscales namely *COVID-19 Anxiety* and *self-care behaviors*, these two factors explained about the half of total variance of anxiety syndrome among the Iranian general population. Although one of the advantages of convenience sampling is that participants are readily available but at a disadvantage, a particular sample may be given more attention or, conversely, a group of the target population may not be included in the sample. Due to the fact that in this study, samples were collected through Google Form and social networks, people who were not members of social networks could not access the data collection form.

In the present study, the high level of Cronbach's alpha (>0.72), McDonald's omega (>0.72), and the average correlation between the items demonstrated that two factors of the scale had acceptable internal consistency. The results of internal consistency were almost similar to the original scale. The calculation of McDonald's omega is the advantage of this study because it does not depend on sample size and numbers of items. Also, based on the results of CR (>0.85) and Max-R, the Persian version of C-19ASS had good reliability. The CR is estimated by factor loading in CFA (26).

Based on the EFA results, two factors were identified, the first of which is *COVID-19 anxiety* which consists of five items. Anxiety is a mental disorder defined by excessive anxiety that leads to panic and is often accompanied by physical symptoms (27). People usually need more information about critical events to reduce anxiety caused by uncertainty in a critical event (27). For this reason, in examining COVID-19 anxiety syndrome in this study, people more than anything, are searching for the signs and symptoms of COVID-19 disease and news related to COVID-19.

The second factor is *self-care behaviors*. Behaviors that are performed with the aim of preventing disease and maintaining individual well-being is commonly defined as self-care behaviors. Self-care is a decision-making process with the aim of preventing COVID-19 and maintaining well-being in the COVID-19 pandemic (28).

The numbers of factors and items of the Persian version of C-19ASS were similar to the original. The only difference between the original version and the Persian version was an item “4) I have been concerned about not having adhered strictly to social distancing guidelines for COVID-19”. This item in the original version loaded into the first factor. But in the present study replaced in the second factor.

The findings of this study indicate that the Iranian version of the C-19ASS scale for evaluating COVID-19 anxiety syndrome is effective and useful in the general population to determine the prevalence of COVID-19. This scale helps the health-care providers, psychologists, and psychiatrists to identify and screen high-risk individuals and to offer preventive interventions to minimize the development of irreversible complications of anxiety syndrome.

The crisis of COVID-19 prevalence in the world, as well as Iran, caused mental disorders and COVID-19 anxiety syndrome, physical, psychological, and financial impacts on people and the government (29). The psychometric analysis of C-19ASS in the Iranian population in this situation showed that the concept of anxiety caused by COVID-19 was explained nearly 50% by the C-19ASS, which contained acceptable psychometric properties.

One of the limitations of this study was related to convenience sampling which is limited in its ability to reach all groups of the population (for example, the elderly population and individuals with no internet or without access to social media such as WhatsApp, Telegram, or email). Since the elderly are more affected by the COVID-19 pandemic due to their vulnerability and it was difficult to access them through social networks, it is recommended that this group be considered in evaluating the scale.

## Conclusion

The Persian version of the C-19ASS scale had an acceptable construct validity and reliability. It has two factors with nine items that explained 48.70% of the total variance of the C-19ASS in the Iranian population during the COVID-19 pandemic. This scale could be beneficial for researchers, psychologists, and healthcare providers to assess anxiety syndrome during the COVID-19 pandemic.

## Data Availability Statement

The data that support the findings of this study are available from the corresponding author upon reasonable request.

## Ethics Statement

The Tehran Islamic Azad University of Medical Sciences Research Ethics Committee approved the protocol of this study (IR.IAU.TMU.REC.1400.315).

## Consent To Participate

Informed consent was obtained from all individual participants included in the study.

## Author Contributions

AE, HS, ES, and EH contributed to the study conception and design. Material preparation and data collection were performed by EH and HS performed data analysis. The first draft of the manuscript was written by EH, PR, HS, and AE. All authors commented on previous versions of the manuscript. All authors read and approved the final manuscript.

## Conflict of Interest

The authors declare that the research was conducted in the absence of any commercial or financial relationships that could be construed as a potential conflict of interest.

## Publisher's Note

All claims expressed in this article are solely those of the authors and do not necessarily represent those of their affiliated organizations, or those of the publisher, the editors and the reviewers. Any product that may be evaluated in this article, or claim that may be made by its manufacturer, is not guaranteed or endorsed by the publisher.
